# Prospective Clinical Feasibility Study for MRI-Only Brain Radiotherapy

**DOI:** 10.3389/fonc.2021.812643

**Published:** 2022-01-10

**Authors:** Minna Lerner, Joakim Medin, Christian Jamtheim Gustafsson, Sara Alkner, Lars E. Olsson

**Affiliations:** ^1^ Department of Hematology, Oncology, and Radiation Physics, Skåne University Hospital, Lund, Sweden; ^2^ Department of Translational Medicine, Medical Radiation Physics, Lund University, Malmö, Sweden; ^3^ Department of Medical Radiation Physics, Clinical Sciences, Lund, Lund University, Lund, Sweden; ^4^ Department of Clinical Sciences Lund, Oncology and Pathology, Lund University, Lund, Sweden

**Keywords:** MRI-only, implementation, brain, glioma, sCT, radiotherapy, cancer

## Abstract

**Objectives:**

MRI-only radiotherapy (RT) provides a workflow to decrease the geometric uncertainty introduced by the image registration process between MRI and CT data and to streamline the RT planning. Despite the recent availability of validated synthetic CT (sCT) methods for the head region, there are no clinical implementations reported for brain tumors. Based on a preceding validation study of sCT, this study aims to investigate MRI-only brain RT through a prospective clinical feasibility study with endpoints for dosimetry and patient setup.

**Material and Methods:**

Twenty-one glioma patients were included. MRI Dixon images were used to generate sCT images using a CE-marked deep learning-based software. RT treatment plans were generated based on MRI delineated anatomical structures and sCT for absorbed dose calculations. CT scans were acquired but strictly used for sCT quality assurance (QA). Prospective QA was performed prior to MRI-only treatment approval, comparing sCT and CT image characteristics and calculated dose distributions. Additional retrospective analysis of patient positioning and dose distribution gamma evaluation was performed.

**Results:**

Twenty out of 21 patients were treated using the MRI-only workflow. A single patient was excluded due to an MRI artifact caused by a hemostatic substance injected near the target during surgery preceding radiotherapy. All other patients fulfilled the acceptance criteria. Dose deviations in target were within ±1% for all patients in the prospective analysis. Retrospective analysis yielded gamma pass rates (2%, 2 mm) above 99%. Patient positioning using CBCT images was within ± 1 mm for registrations with sCT compared to CT.

**Conclusion:**

We report a successful clinical study of MRI-only brain radiotherapy, conducted using both prospective and retrospective analysis. Synthetic CT images generated using the CE-marked deep learning-based software were clinically robust based on endpoints for dosimetry and patient positioning.

## 1 Introduction

Radiotherapy (RT) is an important part of treatment for patients with brain malignancies, such as glioma. Traditionally, RT treatment planning is based on images obtained from both computed tomography (CT) and magnetic resonance imaging (MRI), in which case MRI is used primarily to define the tumor and organs at risk (OAR). In recent years a workflow based on MRI without CT imaging has evolved, referred to as MRI-only radiotherapy (1–3). Excluding CT from the workflow enables reduced spatial uncertainties in the final dose plan since the otherwise required image registration between the CT and the MR images is not needed (4, 5). MRI-only radiotherapy also provides a more streamlined workflow which may reduce both time and costs (1). However, the Hounsfield units (HU) containing electron density information for absorbed dose calculations are not directly present in the MR images. To bridge this gap, synthetic CT (sCT) images, generated based on MRI information, are introduced to provide the necessary HU. Many successful sCT generation methods for brain have been presented in the literature, starting from methods which simply assumed a homogeneous attenuation value inside the head (6) to state-of-the-art deep learning-based methods in recent publications (7–12).

MRI-only RT has been presented for treatment of prostate cancer using both in-house developed methods (13) as well as commercial solutions (14–16). For brain lesions on the other hand, the first commercially available sCT generation products were only recently released on the market (8, 17, 18). Despite the number of previously performed validation studies of sCT for brain (19), this will, to the best of our knowledge, be the first publication on a prospective clinical implementation of MRI-only RT for brain tumors.

In a recent publication by our group (8), a CE-marked sCT generation software was validated in patients with brain malignancies. Results demonstrated equivalent dose distributions and patient treatment positioning between CT and sCT based RT workflows. This work was the foundation and motivation for the present study, using the same sCT generation method in our clinic. To facilitate the implementation of MRI-only RT planning for brain tumors, this study aimed to introduce a new workflow in our clinic based on solely MR images. For quality assurance (QA) purposes only, CT was still acquired to enable both prospective and retrospective analysis.

## 2 Method

### 2.1 Patients and Imaging

In this prospective treatment study 21 glioma patients were consecutively included during March 2020 to March 2021. The study was approved by the regional ethical review board and informed consent was obtained from all patients. Patient details are presented in **Table 1**. Patients above 18 years old referred to CT and MR examinations for treatment planning prior to RT of high-grade glioma were asked to participate in the study. Tumor classification was performed within clinical routine using the WHO 2016 classification of glioma. Study exclusion criteria were any MRI contraindications or metal implants near the tumor. Standardized fractionation schemes with total doses of 34.0, 40.05 or 60.0 Gy (10, 15 or 30 fractions, respectively) were prescribed according to clinical routines, based on tumor malignancy and patient specific factors such as age and comorbidity.

**Table 1:** Patient details.

Patient detail	
Age, mean [range]	62 years [46–85 years]
Gender	12 male/9 female
Diagnosis	Glioma, grade III (n=2)/grade IV (n=19)
Prescribed dose	34.0 Gy, 10 fractions (n=4)
	40.05 Gy, 15 fractions (n=4)
	60.0 Gy, 30 fractions (n=13)
PTV volume, mean [range]	293 cc [139–644 cc]

The proposed MRI-only workflow was inspired by previously published work for prostate cancer (14), appropriately adjusted for glioma. All imaging was performed in treatment setup, using individual three-point fixation masks (Orfit Industries NV, Wijnegem, Belgium) and head support. All patients underwent both CT and MRI examinations, where the CT scan was solely used for QA purposes and not included in any decision making prior to the approval of the treatment plan.

#### 2.1.1 MRI Examination

**Figure 1:**
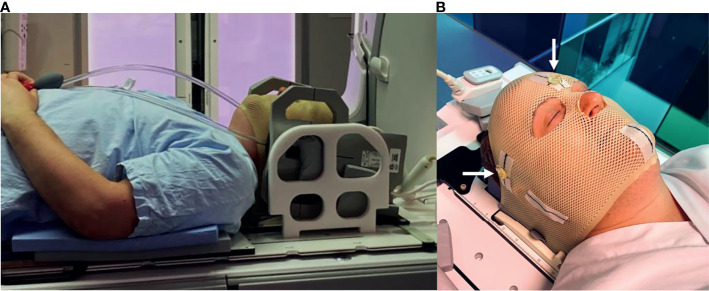
**(A)** RT-setup of patient in three-point fixation mask scanned on a flat tabletop with 6-channel receiver flex coils (left and right) combined with an 8-channel posterior array (under the flat tabletop). **(B)** Fixation mask with liquid markers front, left and right, indicated by the white arrows.

MRI was performed on a 3T GE Discovery 750 W (software release: DV26.0-R03-1831.b, GE Healthcare, Chicago, Illinois, USA) for the first 16 patients and on a 3T GE Architect (software release: DV28.0-R05-2034.a) for the remaining five patients. RT-setup was used during all examinations, including a laser bridge (LAP GmbH Laser Applikationen, Lüneburg, Germany), a flat tabletop and 6-channel receiver flex coils combined with an 8-channel posterior array (**Figure 1A**). Three conical liquid markers (Beekly Medical, Bristol, CT, United States) were placed left, right and front on the fixation mask (**Figure 1B**), according to the laser intersection points, to define the user origin in the images.

MRI sequences for sCT generation and treatment couch identification were added to the clinical brain MRI protocol, as described in previous work (8). A 3D IDEAL Dixon fast spoiled gradient echo (FSPGR) acquisition sequence was used for sCT generation. Slice thickness was 2 mm, in-plane resolution was 1.1x1.1 mm^2^ and scan time was 4.5 minutes. To minimize geometric distortion, the bandwidth was 744 Hz/pixel with 3D distortion correction enabled. Geometric distortions have previously been investigated on the current scanner using the same patient setup and Dixon sequence and were found to be of no clinical concern (8). The resolution after reconstruction of the Dixon images (fat, water, in-phase and out-of-phase) was 0.5x0.5x2 mm^3^. Since the treatment couch did not generate any useful MR signal in the Dixon sequence, a zero-echo time (ZTE) sequence with a total scan time of 21 s was added to image the position of the couch. The acquisition of images for target delineation with and without gadolinium (Gd) contrast agent were included from the standard clinical brain MRI protocol (T1-, T2- and diffusion-weighted images). Total scan time during the whole examination was approximately 25 minutes. Visual inspection of the alignment between images from different MRI sequences was performed after importing the images to the treatment planning system (TPS) by experienced MRI staff.

MRI scanner performance was assessed by monthly quality assurance measurements, as part of our normal clinical routines. These controls included MRI system specific geometric distortion checks with a large field of view phantom (GRADE, Spectronic Medical AB, Helsingborg, Sweden). The phantom contained approximately 1200 signal markers, which were automatically compared to a reference template in the evaluation of geometric distortion.

#### 2.1.2 Synthetic CT Generation

The sCT images were generated using the CE approved sCT generation software MRI Planner (v 2.2, Spectronic Medical, AB, Helsingborg, Sweden), previously validated for both brain and head and neck cancer (8, 20). The software is deep learning-based and utilizes a 3D deep convolutional neural network to generate sCT images based on Dixon images (fat, water, in-phase and out-of-phase). Clinical workflow integration was facilitated by an MRI console DICOM export of the Dixon images to the cloud-based MRI Planner software from which the sCT images were automatically returned to the TPS. The returned sCT images inherited the MR image frame of reference and the same spatial resolution as the reconstructed Dixon images (0.5x0.5x2.0 mm^3^). The liquid markers placed front, left and right on the patient during MRI examination were visible in the sCT images.

#### 2.1.3 CT Examination

CT imaging was performed using a Siemens Somatom Definition AS+ (Siemens, Erlangen, Germany) with 2 mm slice thickness, in-plane resolution between 0.7x0.7 mm^2^ and 1.0x1.0 mm^2^ and tube voltage 120 kV. Although the CT examination was performed prior to MRI examination due to logistic reasons in our clinical workflow, the CT images were not imported to the TPS until the dose plan was completed. Hence, the CT images could in no way influence the target delineation, the treatment planning nor the image registration during treatment positioning as the CT images were strictly used for QA purposes and in retrospective analysis.

### 2.2 MRI-Only Treatment Planning, Approval and Delivery

#### 2.2.1 MRI-Only Treatment Planning

All steps of treatment planning were performed in Eclipse (v 15.6.05, Varian Medical Systems, Palo Alto, CA, USA). Target and organs at risk (OAR) were delineated on MR images overlayed on sCT. The contours of the body and the brain were automatically generated for the sCT images in the TPS, according to clinical routine. The position of the treatment couch relative to the fixation device was identified using the ZTE images and was inserted as a structure in the sCT. This enabled the couch to be accounted for in the optimization and dose calculation. The user origin was set based on the projection of the three liquid markers in the sCT. Finally, treatment plans were created and optimized directly on sCT images, following local clinical routines for high-grade gliomas. All patients were treated using volumetric modulated arc therapy (VMAT) on TrueBeam (Varian Medical Systems, Palo Alto, CA, USA), with two or three arcs. Dose calculation was performed using the standard Eclipse HU calibration curve, also provided by MRI Planner, and an analytical anisotropic algorithm (v. 15.6.05, Varian Medical Systems, Palo Alto, CA, USA) with a 1x1 mm^2^ or 2.5x2.5 mm^2^ dose grid, depending on the target size.

#### 2.2.2 Treatment Plan Approval

All treatment plans based on the sCT were reviewed and approved by experienced oncologists and medical physicists, according to local clinical criteria (**Table E1**, in the **Supplementary Material**). Final treatment approval was performed after finishing the prospective quality assurance steps, see *Prospective QA*.

The study patients were monitored using a logbook attached to each patient’s treatment plan. Notes regarding target delineation, appearance of bone structure and bone resection areas, dose deviations and HU agreement were made by the involved dosimetrist, oncologist and medical physicist. The aim of the logbook was to monitor potential issues during the process.

#### 2.2.3 Treatment Delivery

After treatment approval of the MRI-only plan, it was measured using a Delta4 Phantom+ (Scandidos AB, Uppsala, Sweden) according to local clinical routines. Planned and measured dose were compared using global gamma evaluation with at least 95% of the points passing the criteria 3%, 2 mm required for approval.

Imaging protocol followed clinical routine, which included CBCT imaging the first three treatment fractions and once a week for the remaining fractions. The sCT was used as the image reference in the automatic registration based on mutual information of the bony anatomy.

### 2.3 Quality Assurance

To ensure a safe implementation of the MRI-only workflow, several prospective quality assurance (QA) steps (**Table 2**) were introduced prior to final acceptance of the treatment plan. These included evaluating dosimetry and sCT image quality. Tasks for QA approval were integrated in the TPS, requiring manual confirmation before it was possible to proceed in the workflow. The TPS tasks concerned imaging, post imaging, QA and treatment delivery. Additional analysis of the absorbed dose and patient positioning was performed retrospectively. Comparisons were made against CT in all QA steps, as it is the gold standard imaging modality in RT.

**Table 2:** Summary of the QA steps introduced for MRI-only implementation, including both prospective and retrospective analysis.

QA step	Control	Acceptance criteria
**Prospective QA**		
MRI acquisition parameters script	Automatic control of essential MRI Dixon acquisition parameters against a predefined template	MRI acquisition parameters should be identical to template
Visual inspection	Check sCT for artifacts, verify alignment between MRI sequences	Qualitative evaluation
HU units	Compare HU line profile between sCT and CT	Qualitative evaluation
Bone structures	Check bone structures and bone resection areas to verify correct generation of sCT compared to CT	≤1.5 mm for bone edges
Dose distribution	Recalculate sCT treatment plan on CT and evaluate DVH parameters for target and OARs	i) Dose difference within ±1% for relevant target and OARs or ii) OAR absolute dose more than 10% below clinical tolerance
**Retrospective QA**		
Patient positioning	Verify after treatment start that CBCT registration is equivalent using sCT and CT as reference	≤ 1 mm in x, y and z, respectively

#### 2.3.1 Prospective QA

The first QA step was an automatic MATLAB (v. 2015b, Mathworks Inc., Natick, MA, USA) script developed to check the MRI acquisition parameters of the Dixon sequence. Source code is available at https://github.com/jamtheim/MRIAcqParameterCheckBrain. The parameters were checked against a predefined template to ensure consistent image acquisition throughout the study. A patient specific report of the result was generated and automatically sent by e-mail to the study coordinators for each patient.

The sCT was visually inspected in connection to the target delineation for any artifacts which might have an influence on the treatment before being forwarded to dose planning. After the MRI-only treatment plan had been approved at the ordinary chart round, the CT images were imported to the TPS for QA procedures. The CT was rigidly registered to the sCT images, including translation and rotations for optimal agreement between structures, using automatic bone match (threshold 200-1700 HU). All structures, except for the body contour, were transferred to the CT. A new body contour was automatically generated.

The original treatment plan was recalculated on the CT keeping the same number of monitor units. Due to intrinsic properties, the TPS does not support rotations of the dose matrix, which resulted in a translational registration only of the dose matrix. Evaluations of dose volume histogram (DVH) parameters were performed within the TPS to mimic the conventional workflow. All dose differences were normalized to prescribed dose. Treatment plans were approved without further investigation if all dose differences were within ±1%, comparing sCT and CT based dose calculations. Targets were evaluated based on mean dose (D_mean_), near minimum dose (D_98%_ and D_95%_) and near maximum dose (D_2%_). Two acceptance criteria were used for OARs; i) when the OAR was close to the high-dose region the difference for D_2%_ should be within ±1%, and ii) when the absorbed dose to the OAR was more than 10% below its tolerance dose, only a note of the dose difference was made provided it was below tolerance in absolute numbers including the deviation.

The general appearance of HU line profiles was qualitatively compared between sCT and CT images in the TPS. Bone structures, and especially areas of bone resection due to pre-RT surgery, were inspected near the target. Acceptance criteria of the sCT to CT difference in bone edges and bone resection areas were maximum 1.5 mm.

#### 2.3.2 Retrospective QA

Patient positioning was evaluated through retrospective QA, where the sCT and CT registrations of the CBCT from one of the first three treatment fractions were compared. Acceptance criteria was less than 1 mm difference in any translational direction.

#### 2.3.3 Additional Dose Evaluation

The CT-based dose distribution was corrected for differences in image rotation and image resolution for further analysis. This was performed by rigidly registering and resampling the CT to the sCT frame of reference using the translation and rotation parameters from the TPS in the software MICE Toolkit (Nonpi Medical, Umeå, Sweden). The corrected CT was then imported back into the TPS and the sCT-based treatment plan was transferred and evaluated. This procedure was not found optimal for the clinical workflow and was therefore only used in the retrospective analysis.

In addition, retrospective 3D global gamma evaluation of the sCT and CT calculated dose distributions (>15% of prescribed dose) was performed in MICE Toolkit for all patients. Gamma criteria with the following dose difference/distance to agreement were considered: 1%/1 mm, 2%/2 mm and 3%/3 mm.

## 3 Results

### 3.1 Prospective QA

Twenty out of twenty-one patients successfully received MRI-only RT according to the study workflow. No deviations were found in the automatic MRI acquisition parameters script control. MRI system specific geometric distortions of the MRI scanner were acceptable and stable during the inclusion period (**Table 3**).

**Table 3:** Geometric distortion measured using the Spectronic GRADE phantom, presented as the average of the monthly individual mean and maximum distortions for each given radial distance from the MRI scanner isocenter.

Geometric distortions [mm]			
**Radial distance from isocenter [mm]**	<100	100–150	150–200
**Average of mean distortion (1 SD) [range]**	0.2 (0.1) [0.1–0.4]	0.3 (0.1) [0.2–0.5]	0.6 (0.1) [0.5–0.8]
**Average of max distortion (1 SD) [range]**	0.6 (0.1) [0.4–0.8]	0.9 (0.2) [0.6–1.3]	1.8 (0.2) [1.4–2.2]

Exclusion of a single patient was due to a hemostatic substance injected during pre-RT surgery. The substance gave rise to a signal loss in the MR images which the sCT generation software interpreted as bone. This resulted in up to 5 mm thicker skull bone adjacent to the target in the generated sCT image compared to the CT. The position of the target was temporo-occipital. The patient was excluded from the study as a study precaution, although no clinically significant dose difference or patient positioning effect was observed in a retrospective analysis. This patient was successfully transferred back to the conventional workflow with CT and MRI, with no delay in the scheduled treatment delivery.

**Figure 2:**
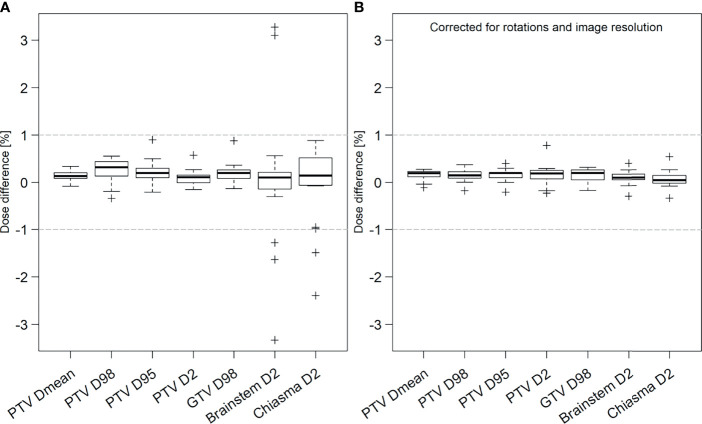
Prospective **(A)** and retrospective **(B)** analysis of dose difference between treatment plans calculated on sCT and CT images for target (PTV and GTV) and organs at risk (OAR) brainstem and chiasma for all patients. In the prospective results, OAR outliers outside ±1% had relative dose levels more than 10% below the clinical tolerance for absorbed dose to OARs. In the additional retrospective analysis, original dose distribution has been corrected for image rotations and image resolution differences between the sCT and CT. The thick black line in each box represents the median value for all patients. The box includes the 25th-75th percentiles, the interquartile range (IQR). Whiskers represent the maximum and minimum values within 1.5 IQR and the crosses represent any values outside that range. The grey horizontal lines represent ±1% dose difference.

Clinical acceptance criteria were fulfilled for all patients receiving MRI-only RT. The target dose parameters were within ±1%, comparing the dose calculated on sCT and CT images (**Figure 2A**). Seven outliers with dose differences outside ±1% were observed for brainstem and chiasma D_2%_. All of these concerned lower dose regions more than 10% below clinical tolerance dose, thus passing the second criteria.

### 3.2 Retrospective QA

**Figure 3:**
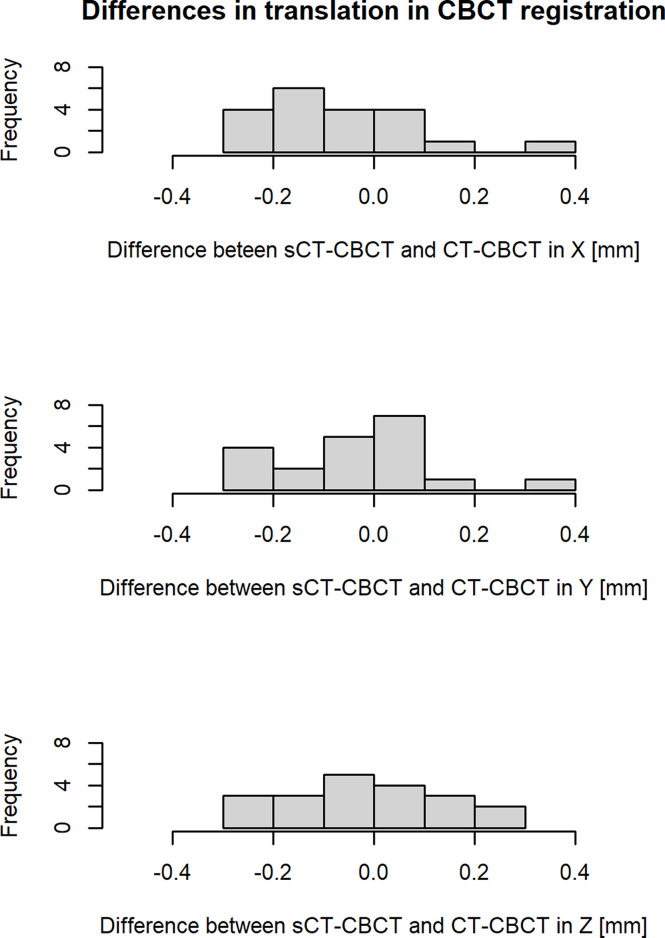
Differences in translation (X, Y, Z) in the image registration of sCT-CBCT compared to CT-CBCT for all patients. The X, Y and Z axis correspond to the following translations: X = left to right, Y = anterior to posterior and Z = superior to inferior. The histogram cells include their right-hand endpoint.

Results from retrospective analysis of patient positioning using CBCT is presented in **Figure 3**. The difference between CBCT registered to sCT and CT was found to be on sub-mm level for all patients and translational directions. The mean ± 1 S.D. (range) 3D vector magnitude of the total registration differences for all patients was 0.3 ± 0.1 mm (0.1-0.6 mm).

### 3.3 Additional Dose Evaluation

When taking rotations and image resolution into account in the retrospective analysis of dose differences, all values were within ±1% (**Figure 2B**).

Global gamma pass rates, comparing the dose distributions calculated on sCT to CT, with a dose cut-off at 15% of the prescribed dose is presented in **Table 4**. For gamma criteria 2%, 2 mm all patients had a gamma pass rate above 99%.

**Table 4:** Global gamma evaluation, with a dose cut-off at 15%, comparing sCT and CT dose distributions, averaged over all patients.

Gamma criteria	Gamma pass rate ± 1 SD [range] (%)
3%, 3 mm	100.0 ± 0.1 [99.7–100.0]
2%, 2 mm	99.8 ± 0.2 [99.4–100.0]
1%, 1 mm	99.1 ± 0.6 [97.6–99.8]

## 4 Discussion

We report the first MRI-only RT treatment study for brain tumors, using a deep learning-based software for sCT generation. The workflow was successfully implemented in the clinic with 20 out of 21 patients receiving MRI-only brain RT. The study was prospective with all treatments optimized, calculated and delivered using sCT images.

All patients receiving the MRI-only treatment passed the prospective acceptance criteria. The TPS tasks regarding imaging, post imaging, QA and treatment delivery were successfully completed for all included patients. Dose differences were within ±1% for both target and OARs, if rotations between the sCT and CT frame of reference were taken into account. CBCT registration with sCT and CT images as reference agreed on sub mm level for all included patients. This being the first study on MRI-only brain RT there are no similar prospective studies to compare with. There are however several published implementation studies on MRI-only RT for prostate cancer, where treatment success rates between 87.5-100% (13, 14, 16) are reported.

The accuracy of sCT images generated from deep learning-based methods relies on a variation of relevant image features to be included in the training data of the model. Implants or abnormal anatomy due to surgery are common in patients treated for brain tumors. This might constitute a problem if the anomaly goes beyond image features included in the training data, since the sCT generation software then is unable to interpret the input MR images correctly. Therefore, it is important to visually inspect the resulting sCT images to find potential artifacts. The only excluded patient of this study was successfully transferred back to the conventional workflow, receiving treatment without any delays. If similar cases would occur during MRI-only RT in clinical routine, the artifact needs to be individually assessed based on its magnitude and localization relative to target and critical anatomical structures. During the implementation phase of MRI-only RT, irrespective of anatomical region, occasional exclusions may be necessary. Since MRI-only workflow implementations lack well-established, simple QA-methods for a safe assessment, a conversion back to the combined CT and MRI based workflow should be accessible during the implementation phase.

This study was a clinical implementation of MRI-only RT. Therefore, we aimed to perform all evaluations in the clinical systems before treatment approval. The TPS used at our clinic only allows translational registrations of dose matrices even if the images are matched using both translations and rotations. This limitation resulted in dose differences above 1% for OARs in six patients due to the OARs being located in low dose regions adjacent to steep dose gradients. However, in addition to relative dose difference, the acceptance criteria for OARs included a comparison with clinical tolerance (as described in section 2.3.1). These six patients passed the second criteria and received their treatment based solely on the sCT. In addition, the patients with dose deviations above 1% in this study were analyzed retrospectively applying both translations and rotations of the dose matrices (as described in section 2.3.3) and were all found to be well within 1%. The rationale for applying a limit of 1% in dose differences in clinically relevant DVH parameters between the sCT and the CT based treatment plans is based on a goal to achieve less than 2% systematic dose error in the delivered treatment. It has been shown that a reasonable accuracy to strive for in systematic bias in dose delivery is 1-2%, taking tumor response and normal tissue response into account (21). In this context one must consider that there are several potential systematic biases/errors in the chain from imaging to treatment delivery which contributes to the final error, for example inherent limitations in dose calculation algorithms and treatment machine calibration. Although 2% has been suggested as acceptance criteria for different anatomies within MRI-only implementations previously (22), we suggest that a limit of 1% might be appropriate for MRI-only brain RT. This is further supported in recent publications using deep learning-based sCT generation methods (8–12). A 1% criteria on sCT-CT dose difference may still enable a total bias/error in delivered dose below 2% after the contribution from other systematic errors. Furthermore, the recommended limit for random uncertainties is less than 3% (21), which also needs to be added to the systematic uncertainties discussed above.

During the implementation of MRI-only RT two aspects of the dose criteria should be considered; 1) relative dose difference between sCT and CT based calculations and 2) absolute dose level compared to clinical tolerance for OAR in low dose regions. To establish general acceptance criteria for MRI-only brain RT implementations, more and larger prospective studies are required. Until then, each clinic needs to perform their own studies as part of their implementation.

In a recent review (23) recommendations for several deep learning-based applications in radiotherapy were summarized. Specifically, regarding the implementation process of sCT generation software, Vandewinckele et al. emphasized the need for user knowledge to be able to detect artifacts and identify their causes. As seen in this study, deviations from the characteristics in the training data set can cause artifacts such as abnormal bone structures. Although some artifacts might be difficult to identify, those are unlikely to have any clinical relevance as dose calculations are relatively insensitive to small HU variations. There is however still a need for case specific QA. One suggestion could be to use CBCT for dose calculation as an independent evaluation of the sCT, which would likely find most clinically relevant deviations (23–25). Regular sCT generation model QA is another important aspect of implementing a deep learning-based software. This is especially important if changes are made to the workflow, such as modifications of MRI acquisition protocols or MRI scanner hardware. During the present study, the MRI scanner was upgraded from a GE Discovery (software DV26) to a GE Architect (software DV28). Monthly QA routines verified that the MRI scanner was stable regarding geometric distortion, before and after the upgrade. Minor changes in sCT characteristics were however observed, manifested as streaks of slightly higher HU values in cranial parts of the brain. This was due to minor changes in the MRI scanner post processing but had no dosimetric impact for this patient cohort. Despite QA for both geometry and MRI acquisition parameters, this was not captured until visual inspection of the sCT.

To summarize, implementing a new workflow in the clinic can be challenging. However, the transition can be done safely by making conscious and careful changes in all steps of the workflow, with thorough validation studies, appropriate QA and close collaboration with the clinical staff.

## 5 Conclusion

In this prospective clinical MRI-only RT study for brain tumors, an implementation of a commercial deep learning-based sCT generation method was conducted using both prospective and retrospective analysis. The workflow was successfully applied to 20 glioma patients, fulfilling both dosimetric and treatment setup criteria.

## Data Availability Statement

The datasets presented in this article are not readily available because of patient privacy concerns and institutional regulations. Requests to access the datasets should be directed to ML, minna.lerner@med.lu.se.

## Ethics Statement

The studies involving human participants were reviewed and approved by Regional ethical review board, Lund, Sweden DNR: 2018/445. The patients/participants provided their written informed consent to participate in this study.

## Author Contributions

Conceptualization: JM, SA, and LO. Patient recruitment: ML, JM, and SA. Clinical guidance: JM and SA. Data analysis: ML. Software: ML and CJ. Drafting manuscript, review and editing: ML. Proof editing: JM, CJ, SA, and LO. All authors approved the submitted version.

## Funding

This work was supported by “Allmänna sjukhusets I Malmö Stiftelse för bekämpande av cancer”; Fru Berta Kamprads stiftelse för utforskning och bekämpning av cancersjukdomar, SUS foundations and Region Skåne.

## Conflict of Interest

The authors declare that the research was conducted in the absence of any commercial or financial relationships that could be construed as a potential conflict of interest.

## Publisher’s Note

All claims expressed in this article are solely those of the authors and do not necessarily represent those of their affiliated organizations, or those of the publisher, the editors and the reviewers. Any product that may be evaluated in this article, or claim that may be made by its manufacturer, is not guaranteed or endorsed by the publisher.
