# Supplementary material for: Prospective Clinical Feasibility Study for MRI-Only Brain Radiotherapy
Source: Front Oncol. 2022 Jan 10;11:812643. doi: 10.3389/fonc.2021.812643 (PMC8784680; doi:10.3389/fonc.2021.812643)
Supplement: Supplementary file 1 [file Table_1.docx]

| **Prescribed dose** | | | **60.0 Gy** | | **40.05 Gy** | **34.0 Gy** |
| --- | --- | --- | --- | --- | --- | --- |
| **Priority** | **Structure** | **DVH criteria** | **[Gy or % of prescribed dose]** | | | |
| 1 | Chiasm / optic nerves | D_2%_≤ | 54 | 40.05 | | 34 |
| 2 | PRV chiasm / PRV optic nerves | D_2%_≤ | 56 | 40.05 | | 34 |
| 3 | Brainstem | D_2%_≤  V_54Gy_≤ | 60  30% | 40.05  - | | 34  - |
| 4 | PRV brainstem | D_2%_≤ | 60 | 40.05 | | 34 |
| 5 | GTV | D_98%_≥ | 97% | | | |
| 6 | PTV | D_98%_≥  D_95%_≥ | 95%  93% | | | |
| 8 | Retina | D_2%_≤ | 45 | 37 | | 33 |
| 9 | Lenses | D_2%_≤ | 5 | 5 | | 5 |
| 10 | Cochlea | D_mean_≤ | 40 | 33 | | 32 |
| 11 | Pituitary | D_mean_≤ | 50 | 40.05 | | 34 |
| 12 | Lacrimal gland | D_mean_≤ | 35 | 30 | | 24 |
| 13 | Hippocampus | D_40%_≤ | 7 | 5 | | 5 |

**Table E1.** Dose volume constraints and objectives for clinical acceptance used in this study, presented for three levels of prescribed dose; 60.0, 40.05 and 34.0 Gy.

Abbreviations: DVH: Dose Volume Histogram, PRV: Planning organ at Risk Volume, GTV: Gross Tumor Volume, PTV: Planning Target Volume
